# Supplementary figures and images for: Mental disorder recovery correlated with centralities and interactions on an online social network
Source: PeerJ. 2015 Aug 20;3:e1163. doi: 10.7717/peerj.1163 (PMC4548489; doi:10.7717/peerj.1163)

# The Distribution of Recovery Outcomes in Study I

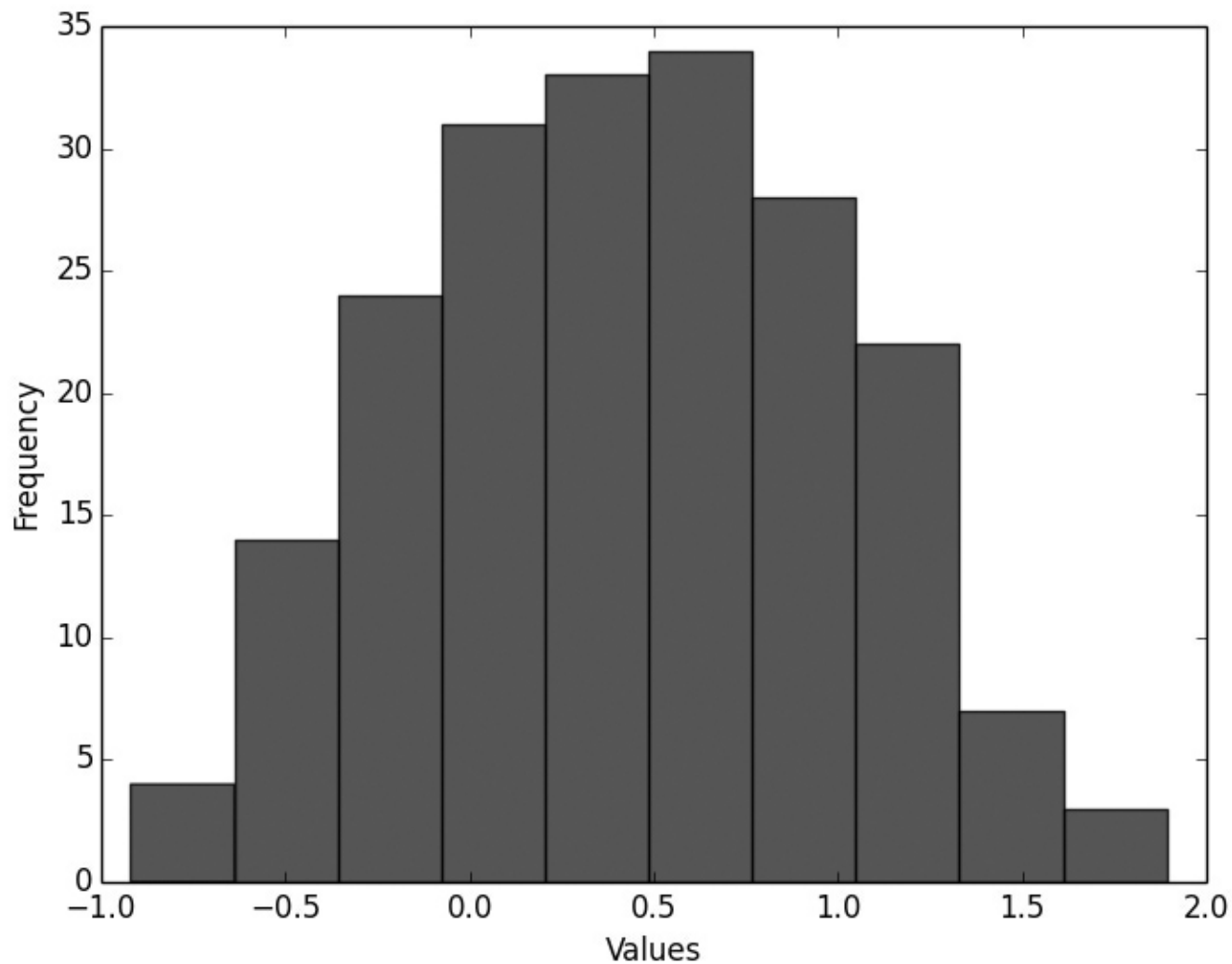

Supplement: Figure S1 [file peerj-03-1163-s001.pdf]

## The Distribution of Recovery Outcomes in Study II

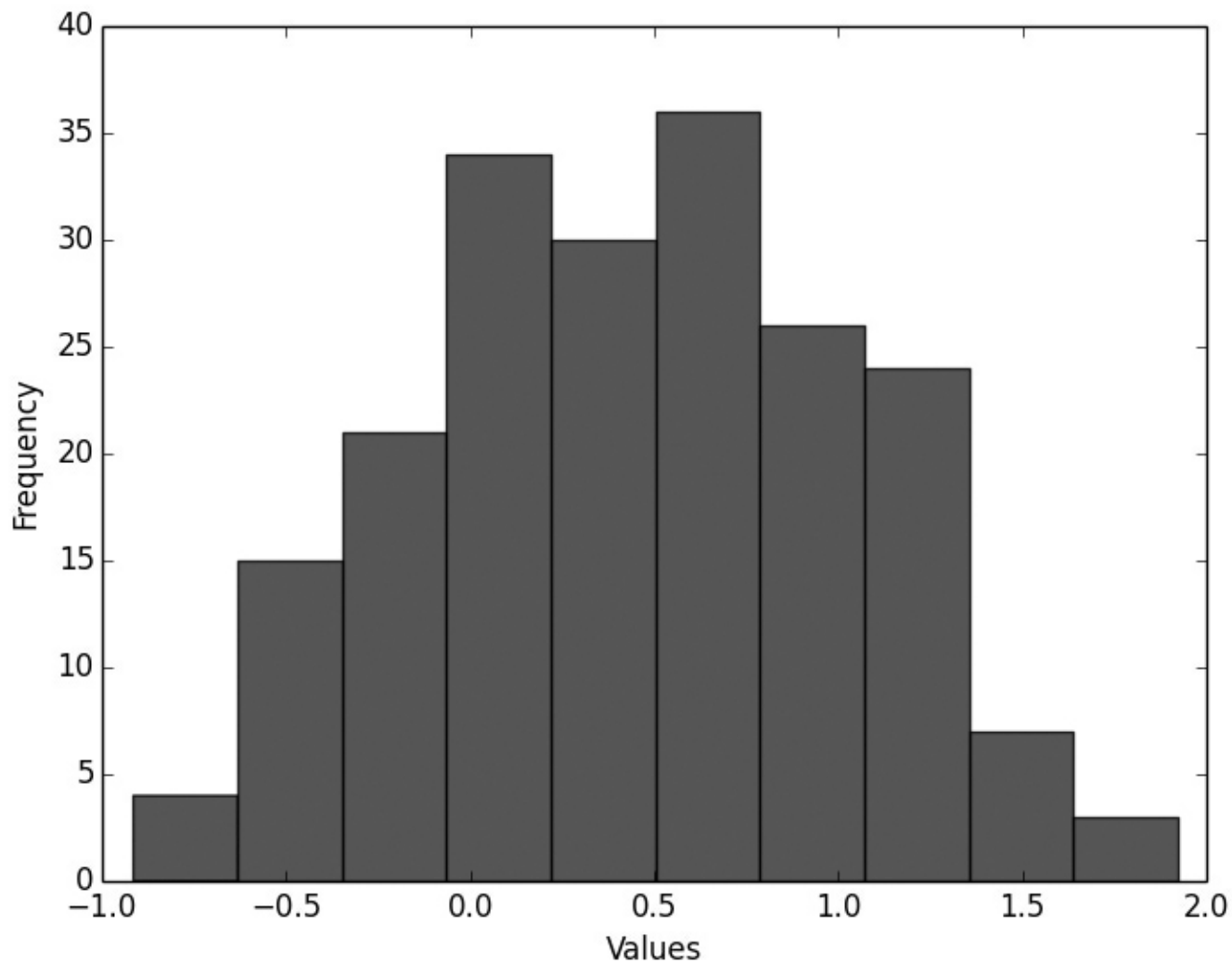

Supplement: Figure S2 [file peerj-03-1163-s002.pdf]
