# Supplementary material for: Mental disorder recovery correlated with centralities and interactions on an online social network
Source: PeerJ. 2015 Aug 20;3:e1163. doi: 10.7717/peerj.1163 (PMC4548489; doi:10.7717/peerj.1163)
Supplement: Table S1 — ∗∗ Correlation is significant at the 0.01 level ∗ Correlation is significant at the 0.05 level [file peerj-03-1163-s003.docx]

| **Correlation** | | **In-degree** | | **Helpful Marks** | | **Posts and Views** | |
| --- | --- | --- | --- | --- | --- | --- | --- |
| **In-degree** | | 1 | |  | |  | |
| **Helpful Marks** | | 0.505** | | 1 | |  | |
| **Posts and Views** | | 0.304** | | 0.590** | | 1 | |
