# Supplementary material for: Mental disorder recovery correlated with centralities and interactions on an online social network
Source: PeerJ. 2015 Aug 20;3:e1163. doi: 10.7717/peerj.1163 (PMC4548489; doi:10.7717/peerj.1163)
Supplement: Table S3 — ∗∗ Correlation is significant at the 0.01 level ∗ Correlation is significant at the 0.05 level [file peerj-03-1163-s005.docx]

| **Recovery Outcome in Study II** | **Mood Function** | **Stress** | **Distress** | **Life Essentials** | **Symptoms** |
| --- | --- | --- | --- | --- | --- |
| **Mood Function** | 1 |  |  |  |  |
| **Stress** | -0.13 | 1 |  |  |  |
| **Distress** | 0.264** | 0.372** | 1 |  |  |
| **Life Essentials** | 0.344** | -0.157 | -0.016 | 1 |  |
| **Symptoms** | 0.206** | 0.042 | -0.03 | 0.301** | 1 |
